# Addressing the Complexities of Physical Health Monitoring in ADHD and Autism in Oxfordshire: Implications for Overall Well-Being and Psychiatric Treatment

**DOI:** 10.1192/bjo.2025.10317

**Published:** 2025-06-20

**Authors:** Andreea Dumitrascu, Sureyya Melike Toparlak, Emma Fergusson, Marta Costa, Robert Chapman

**Affiliations:** Oxford Health NHS Foundation Trust, Oxford, United Kingdom

## Abstract

**Aims:** Individuals with ADHD and autism may face increased risks of cardiovascular issues or metabolic disorders influenced by both their neurodevelopmental traits and prescribed treatments. Ensuring consistent monitoring can help manage these risks and support better long-term outcomes. This paper explores the challenges of physical health monitoring in ADHD and autism and presents a quality improvement project aimed at enhancing monitoring practices in clinical care.

**Methods:** Challenges in physical health monitoring for individuals with ADHD and autism include variability in practice, limited access to medical equipment, space constraints in clinical settings, and the need for clearer guidelines. To address these issues, we conducted an assessment within the ADHD and autism service in Oxfordshire to identify essential materials for comprehensive monitoring of ADHD medications, antipsychotics, and antidepressants, alongside overall physical well-being.

**Results:** Key materials identified included blood pressure monitors, ECG machines, height and weight measurement tools, blood glucose and cholesterol testing kits, liver and kidney function tests, electrolyte testing kits, drug screening tests, and nutritional assessment tools. The assessment identified several challenges in physical health monitoring within ADHD autism service. Out of 12 assessed items, 58.33% had the necessary materials available, though essential equipment was not always present, and time constraints made integration difficult. Among those, 85.7% had functioning equipment, while 14.3% had non-functional equipment.

**Conclusion:** In ADHD and autism services, where psychopharmacology plays a central role in treatment, the importance of physical health monitoring becomes even more critical due to the side effects of medications such as stimulants, antipsychotics, and antidepressants. Inconsistencies in equipment availability, maintenance, and staff training were noted, leading to potential risks to patient safety, reduced efficiency, and increased costs. Recommendations include improved maintenance, acquisition of additional equipment, and enhanced staff training to ensure effective monitoring across services.

